# Relationship between Social Media Use and Social Anxiety in College Students: Mediation Effect of Communication Capacity

**DOI:** 10.3390/ijerph20043657

**Published:** 2023-02-18

**Authors:** Fengxia Lai, Lihong Wang, Jiyin Zhang, Shengnan Shan, Jing Chen, Li Tian

**Affiliations:** 1 Department of Nursing, The First Affiliated Hospital of Soochow University, Suzhou 215006, China; 2 School of Nursing, Medical College of Soochow University, Suzhou 215006, China

**Keywords:** social media use, social anxiety, communication capacity, college students, mediation effect

## Abstract

An increasing number of college students are experiencing social anxiety in an era of prevalent social networking. College students’ social anxiety may be related to their social media use. However, this relationship has not been confirmed. This study aimed to investigate the relationships between different types of social media use and social anxiety among college students, and the mediation effects of communication capacity in this context. A large sample of 1740 students from seven colleges in China was analyzed. Bivariate correlation and structural equations analysis showed that passive social media use was positively correlated with social anxiety. Active social media use was negatively correlated with social anxiety. Communication capacity partially mediated the relationship between social media use (passive/active) and social anxiety. Active social media use may reduce social anxiety by positively mediating communication capacity, while improved communication capacity may reduce the contribution of passive use to social anxiety. The differences in the effects of different social media use on social anxiety deserve the attention of educators. Developing communication capacity education around college students may help reduce their social anxiety.

## 1. Introduction

Social anxiety, also known as “social terror”, refers to the negative anxiety that individuals experience in real or imaginary social interaction situations due to the fear or apprehension of receiving negative evaluations from others [1]. The prevalence of social anxiety in college students is about 7–33% worldwide [2,3,4], while in China, up to 12–14% of college students suffer from high levels of social anxiety [5]. If social anxiety is not corrected or improved, it may develop into a severe social anxiety disorder and continue to affect students’ academic achievement, career development, and mental health [6]. Given the burden that social anxiety places on people and society, it is imperative to study the mechanisms through which it occurs, and to develop interventions.

Simultaneously, the use of social media has increased dramatically over the past decade, particularly among young people. Social network sites (SNSs) such as Instagram, Facebook, and Twitter have become indispensable parts of people’s lives. According to statistics, there are 2.23 billion monthly active Facebook users worldwide, and this figure has an annual growth rate of 11% [7]. In China, the number of internet users had reached 1.011 billion by June 2021, with college students accounting for the highest occupational percentage, at over 23.0%.

Since college students frequently use the internet, their psychological status in social interactions may be influenced by the use of social media. According to research, using SNSs may cause personal social anxiety [8]. Several theories have shed light on possible mechanisms through which social media use triggers social anxiety. According to the self-presentation theory [9], individuals may be more sensitive to negative evaluations of others, and even tend to guess that others have negative evaluations of them in their online self-presentation, which causes social anxiety. Individuals use others as a standard of comparison for self-evaluation in the absence of actual reference material, according to classical social comparison theory [10], especially in the absence of communication, and passive use of SNSs by individuals triggers more upward social comparison. According to behaviorist theory [6], social anxiety is caused by a conditioned reflex of emotional response, implying that social anxiety may be caused by a lack of social skills and, more precisely, communication capacity. 

This evidence calls for a better understanding of the risk factors for social anxiety. These factors also include the way social media is used, interpersonal communication capacity, and previous experiences of social frustration. In this research, we aim to examine the relationship between social media use and social anxiety, taking into consideration the mediating role of communication capacity.

### 1.1. Social Media Use and Social Anxiety

Social media provides an online medium that allows users to add “friends” to the same network and share their personal feelings, photos, etc., with these “friends” [11]. The use of social media makes social comparison easier among young adults, leading to poor mental health and life dissatisfaction [12]. Some studies have found that social media use may trigger social anxiety in individuals. A study conducted in Kolkata discovered that social networking sites (SNSs) and dependence on them had significant associations with anxiety and depression among medical students [13]. Furthermore, according to a Hong Kong, China study, students who spent more time on SNSs had more severe depression and anxiety problems [14]. Users of social media may experience a physiological stress response as a result of receiving negative feedback from others, cyberbullying, becoming more aware of stressful events occurring in the lives of others, and feeling pressure to keep social networks updated [15,16]. Social media use may also lead to general communication overload, as individuals are bombarded with messages from multiple electronic channels at the same time, which is linked to psychological distress [17]. 

Researchers have categorized social media use into active and passive use based on the different ways social networking sites are used [18]. Active social media use is actively communicating with others (posting their news, commenting on friends’ posts, and other information-generating behaviors); passive social media use mainly refers to browsing social networking sites without interacting with others (viewing friends’ news and not participating in comments) [19]. To date, the literature distinguishing the different ways of using social media remains limited. However, a recent study suggests that there may be differences in the impact of different social media uses on individual mental health [20]. Active and passive Facebook use was also found to show opposing effects on loneliness [18]. Based on prior evidence, we came up with the following hypothesis:

**Hypothesis** **1** **(H1):** *Higher active social media use and lower passive social media use are positively associated with lower social anxiety.*

### 1.2. The Mediating Role of Communication Capacity

Communication capacity refers to the ability to receive and transmit information, to effectively and clearly express thoughts, feelings, and opinions to others through written, oral, and nonverbal cues, and to quickly and accurately interpret the information transmitted by others to understand their thoughts, feelings, and attitudes [21]. Multiple lines of evidence suggest that internet use is associated with poorer cognitive-emotional regulation and communication capacity [22,23,24,25]. Furthermore, according to behaviorist theory, social anxiety is caused by a conditioned reflex of emotional response, implying that social anxiety may be caused by a lack of necessary communication capacity [6]. Hence, we decided to focus on communication capacity as a mediator of social anxiety.

While social media users may gain a lot of social contact as a result of their internet use, it decreases face-to-face contact, which may impair their social skills in the real world [23]. Individuals must constantly practice communication skills and modify their behavior in response to feedback from others, and focusing on internet use may limit people’s opportunities to practice communication skills and correct communication capacity deficits [23]. Heavy use of online social networks, for example, has been shown to reduce individuals’ intimacy and time with parents and family, while increasing conflict with people close to them [24]. Adolescents who use social media for extended periods were reported to have more severe social skills deficits [25]. Furthermore, a recent study found that active social media use improves subjective wellbeing, while passive social media use decreases subjective wellbeing [26]. Thus, rather than social media itself, the consequences of social media use may be related to how social media is used. Based on past evidence, we propose the following hypothesis:

**Hypothesis** **2** **(H2):** *Higher active social media use and lower passive social media use are positively associated with better communication capacity.*

The capacity to communicate is also critical in social interaction, as college students must manage relationships with different people, for example, classmates, teachers, romantic partners, and even strangers. Difficulties with effective communication during social development can lead to difficulties in establishing and maintaining friendships, which can result in anxiety, low mood, and depression [27]. Evidence suggests that social anxiety disorder is significantly associated with greater degrees of communication difficulties [25]; in other words, social anxiety is linked to a failure to apply social skills and engage in social interactions [28]. Effective communication skills may affect people’s capacity to cope with worry in their social interactions [29], and social communication deficits may underpin anxiety disorders in individuals suffering from social anxiety. Spending time with others, on the other hand, can improve social skills, but socially anxious people tend to avoid social interactions [14]. Moreover, according to an intervention study, improving social skill knowledge has a positive effect on reducing symptoms of depression and anxiety [30]. Therefore, for the mediation model of this study, in which social media use is directly related to social anxiety and mediated through communication capacity, we hypothesized that:

**Hypothesis** **3** **(H3):** *Better communication capacity is negatively associated with higher social anxiety.*

**Figure 1:**
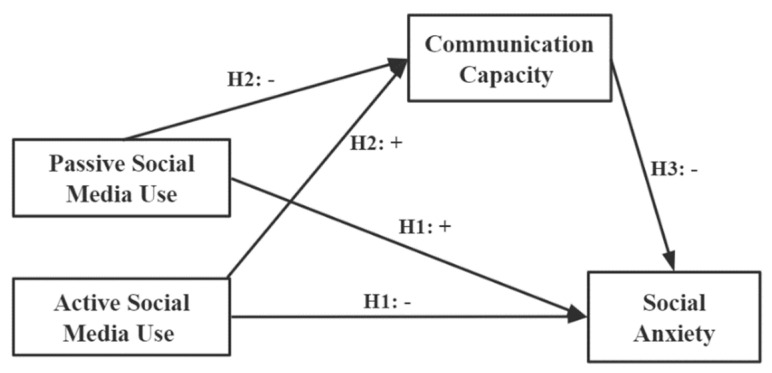
Theoretical mediation model.

The theoretical moderated mediation model is represented in Figure 1.

## 2. Materials and Methods

### 2.1. Study Design and Participants

A cross-sectional design was implemented. This study was conducted from June to September 2022 with students at seven public colleges in Suzhou, China. A total of 2192 college students were surveyed by online questionnaire, and data from 1740 individuals were eventually included through lie detection questions screening (valid recovery rate = 79.4%). Study procedures involving human participants followed institutional ethical standards. All participants completed the questionnaire anonymously after providing informed consent. 

### 2.2. Measurements

All measures were self-reported, and data were obtained via Sojump.com (a platform providing functions equivalent to those of Amazon Mechanical Turk). The questionnaire link was shared by teachers for students to fill out anonymously and could be submitted only once.

#### 2.2.1. Basic Information

Basic information was collected via a general information questionnaire designed by the researchers according to the content of the study, including students’ information such as age, gender, year of study, place of origin, family structure, economic level, and ethnicity. Finally, based on previous studies measuring risk factors related to social anxiety in college students [3,4,5], participants were asked about class leaders’ experiences, frustration experiences in social interactions, their number of friends on SNSs, and childhood left-behind experiences (i.e., those whose parents are migrant workers and those who were children left at home and cared for by relatives).

#### 2.2.2. Active Social Media Use

Active social media use was measured via the Active SNS Use Scale [31]. It mainly measures individuals’ active use of social media, such as “updating information on their social networking site pages” and “posting photos on their social networking site pages.” This scale consists of 5 items. Each item is rated on a 5-point scale (1 = never to 5 = always), with higher total scores indicating higher levels of active social media use. The Cronbach’s α value for this questionnaire is 0.77.

#### 2.2.3. Passive Social Media Use

Passive social media use was measured via the Passive SNS use Scale [32], using a Chinese version revised by Liu Qingqi [33]. It mainly measures individuals’ passive use of social media, such as “reading friends’ status updates” and “viewing photos uploaded by friends”. This scale consists of 4 items. Each item is rated on a 7-point scale (1 = never to 7 = multiple times a day), with higher total scores indicating higher levels of passive social media use. The Cronbach’s α coefficient for this questionnaire is 0.70.

#### 2.2.4. Communication Capacity

Communication capacity was measured via the Communication Capacity Scale developed for college students by a Chinese scholar [34]. This scale consists of 38 items in 8 dimensions: respect, listening, empathy, emotional sensitivity, comforting others, emotional control, enthusiasm, and verbal expression. Each dimension includes 3–7 items (see Appendix A for all scale items); each item is rated on a 5-point scale (1 = not at all to 5 = fully), with higher total scores indicating higher levels of passive social media use. In this study, the Cronbach’s α value of this questionnaire was 0.89.

#### 2.2.5. Social Anxiety

Social anxiety was measured via the Interaction Anxiety Scale [35]. It is mainly used to assess the subjective feelings of individuals’ social anxiety experience and is widely used in related studies. The scale consists of 15 questions and is scored on a 5-point scale (1 = not at all to 5 = fully), with higher total scores indicating higher levels of social anxiety. In this study, the Cronbach’s α value of this questionnaire was 0.81.

#### 2.2.6. Personality Traits

The personality of introversion and extraversion was measured via the Chinese version [36] of the Eysenck Personality Questionnaire [37]. The scale is suitable for Chinese adults aged 16 years and above, with the advantage of being easy to understand and simple to measure. As social anxiety is mainly influenced by introversion and extroversion personality traits in the Chinese ethnic context [38,39], only the E scale with 12 items was used in this study to analyze the influence of introversion and extroversion personality factors on the results. In this study, the Cronbach’s α value of this questionnaire was 0.83.

### 2.3. Statistical Analysis

Descriptive statistics were used to describe participants’ sociodemographic characteristics and the main study variables (age, gender, profession, family structure, the experience of class leaders, etc.). Moreover, we conducted tests of normality and homogeneity of variance. One-way analyses of variance (ANOVAs), *t*-tests, and Pearson’s r correlations were used to test for the unadjusted associations between variables. Because the data obtained are all self-reported by students, this may lead to common method bias (CMBs). Harman’s single-factor test was used to test CMBs. If a factor accounts for more than 50% of the total variance, common method bias has an impact on the findings [40]. All these analyses were performed using IBM SPSS 26.0. Then, to achieve the main objective of the study, a simple mediation model with 5000 bootstraps was run using IBM SPSS Amos 22.0 As an estimate, a 95% confidence interval (CI) was provided. For the mediation effect, a mediator is significant if the 95% CI of the indicator does not include 0 [41]. Confirmatory factor analyses (CFA) were performed and then evaluated by Hu and Bentler’s [42] guide to various fit metrics. The following indicators and thresholds are included: the chi-square/degrees of freedom (χ^2^/*df* < 3.00), the root mean square error of approximation (RMSEA ≤ 0.08), the goodness-of-fit index (GFI > 0.90), and the comparative fit index (CFI ≥ 0.95). All statistical tests were two-tailed, and the level of significance was set at 0.05.

### 2.4. Ethical Considerations

The Institutional review board of Soochow University has approved the ethical considerations in research methods and procedures (SUDA20220620H08).

## 3. Results

### 3.1. Descriptive Statistics

The descriptive statistics for all study variables are shown in Table 1. The mean age of college students was 19 years (standard deviation [SD] = 2), with a range of 15–29 years. 679 (39%) were male students, and 1061 (61%) were female students. The ratios of education levels were 23.0% (undergraduate) and 77.0% (junior college). 57.0% were only children and 43.0% were non-only children; 59.5% had served as student leaders and 40.0% had not. The mean Interaction Anxiety Scale score was (43.46 ± 8.33), indicating a moderate to high level of social anxiety. 

There were significant differences in social anxiety among college students by gender, family income, the experience of being left behind in childhood, frustration experiences in social interactions, and personality traits (Table 1). According to hoc tests, college students with a per capita household income of less than RMB 3000 had higher levels of social anxiety than those with a per capita household income of more than RMB 5000. The lower the income, the higher the level of social anxiety. Students with more frequent experiences of interpersonal frustration had higher levels of social anxiety than those with occasional or no such experiences. Students with introverted personality traits had higher social anxiety levels than those with intermediate and extroverted personality traits. There were no significant differences in social anxiety between the other sociodemographic variables.

### 3.2. Correlations between Social Media Use, Communication Capacity, and Social Anxiety

Pearson’s correlation analysis (Table 2) showed that active social media use was negatively correlated with social anxiety (r = −0.342, *p* < 0.001) and positively correlated with communication capacity (r = 0.514, *p* < 0.01). Passive social media use was positively correlated with social anxiety (r = 0.525, *p* < 0.01) and negatively correlated with communication capacity (r = −0.253, *p* < 0.01). Communication capacity was negatively associated with social anxiety (r = −0.371, *p* < 0.01).

### 3.3. Common Method Bias Test

Harman’s single-factor test showed that a total of 10 factors’ eigenvalues were >1, the interpretation rate of the first factor was 33.47% (<50%), and there was no serious common method bias. 

### 3.4. Model Test

**Figure 2:**
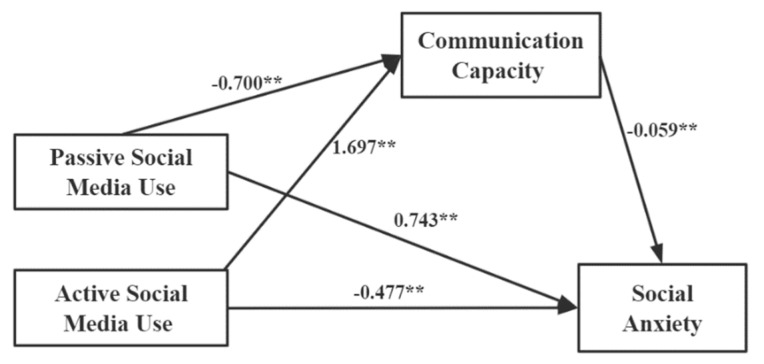
Model of the mediating effect of communication capacity on the association between social media use and social anxiety. Note: ** *p* < 0.01. The normalized path coefficient is represented by the number on the solid line. There are two mediation paths in the diagram: 1. Passive social media use → Communication capacity → Social anxiety 2. Active social media use → Communication capacity → Social anxiety.

We used structural equation modeling with observed variables in SPSS Amos 22.0 to test the relationships between social media use, communication capacity, and social anxiety, while controlling for sociodemographic variables (gender, income, experience of being-left-behind, experience of interpersonal frustration, and personality traits). Figure 2 shows a simplified version of the calculated structural equation model. According to Table 3, the following model can be accepted: χ^2^/*df* = 2.121, CFI = 0.975, GFI = 0.975, AGFI = 0.998, and RMSEA = 0.039. 

The results (Table 4) showed that active and passive social media use had a positive and negative predictive effect on communication capacity (β = 1.697 and −0.700, *p* < 0.01), respectively, explaining 32.8% of the variance in communication capacity. Active and passive social media use negatively predicted social anxiety (β = −0.477 and 0.646, *p* < 0.01) and explained 41.3% of the variance in social anxiety. Bootstrap repeat sampling was set to 5000 and with 95% CI. The results showed that the direct effects were −0.477 and 0.646; the 95% CIs were (−0.552 to −0.401) (*p* < 0.001) and (0.577 to 0.715) (*p* < 0.001). In contrast, the indirect effects were −0.100 and 0.050, and the 95% CIs were (−0.086 to −0.030) (*p* < 0.001) and (0.007 to 0.059) (*p* < 0.001). The study revealed that the direct and indirect effects of social media use on social anxiety are statistically significant; 95% of the CIs did not include zero, which indicated that there was a significant mediation effect of communication capacity on the relationship between social media use and social anxiety.

## 4. Discussion

This study confirmed that higher active social media use and lower passive social media use were associated with lower social anxiety. Furthermore, this relationship was partially mediated by communication capacity.

### 4.1. Social Anxiety Was Affected by Gender, Family Income, the Experience of Being Left behind in Childhood, Frustration Experience in Social Interactions, and Personality Traits

Social anxiety problems developed by college students during their development are often the result of a combination of personal, external, and other factors. Research has documented that sociodemographic variables are crucial factors related to social anxiety [43,44]. In this study, female students perceived more social anxiety than male students. According to self-construction theory [45], men and women have different understandings of self-awareness. Men tend to construct and maintain an independent sense of self in which others are separate from the self. In contrast, women tend to construct an interdependent sense of self in which others are also considered an essential part of the self-construction. This difference in self-awareness may lead female college students to show nervousness and anxiety during social interactions and sensitivity to the evaluation of social partners. They tend to invest time and energy in repeatedly recalling and evaluating their performance after social interactions, and thus are more likely to experience high levels of social anxiety [46]. Moreover, students’ family economic level was associated with social anxiety. Social anxiety was higher among college students with lower monthly per capita household income, which may be related to low self-esteem due to low purchasing power [47]. Similarly, a study by Jefferies [48] on social anxiety among young people in seven countries showed that the unemployed population had higher social anxiety than those employed. 

On the other hand, experiences of being left behind in childhood and interpersonal frustration in social interactions were also associated with higher levels of social anxiety. This triggering mechanism may work through the insecure attachment type of the students [49]. Experiences of traumatic events and adverse life events suffered in childhood have a profound impact on individuals. They become more fearful of interacting with people, fear being judged, have a negative, skeptical attitude toward themselves, and do not participate in as many social activities [50,51]. The findings also showed that extraversion was negatively related to social anxiety; this is consistent with previous studies [50,52]. Highly extroverted people tend to be sociable, talkative, enthusiastic, and confident [53]. These people are more likely to engage in social activities and feel energized by social interactions [54]. Hence, they are less likely to report social anxiety.

### 4.2. Different Manners of Social Media Use Correlated Differently with Social Anxiety and Communication Capacity

Our results showed that the relationship between different use of social media and social anxiety among college students varied. Active social media use was negatively associated with social anxiety, while passive social media use was positively associated with social anxiety. Although the effects were somewhat weak according to the effect size criteria used by Cohen [55], the results still support our research hypothesis 1. In previous studies, less research has been conducted on active social media use, with most of them pointing to positive psychological outcomes [31,56,57]. The reason for this may be related to the fact that active social media use enhances social communication, leading to an increase in daily contact and emotional interaction [18]. Users increase their positive emotions by interacting directly with other users and increasing their supportive interactions online [58], thereby reducing social anxiety. Passive social media use, on the other hand, points to adverse outcomes in this study. Previous research has shown that passive use of social media positively predicts loneliness, leads to a decrease in individual wellbeing, and affects adolescent body image worries [59]. This is because the content presented by individuals on the internet can make the viewers feel that their friends’ life is better than their own, which in turn affects subjective wellbeing [60]. 

Moreover, this study revealed that active social media use was positively associated with the communication capacity of college students, while passive use was the opposite. Active online communication has been shown to have a beneficial effect on individuals. For instance, active social media use can indirectly influence friendship quality through positive SNS feedback, and can positively predict friendship quality [61]. On the other hand, passive social media use may partially replace the function of real-life interactions and also crowd out the real-life interaction time of college students. It may diminish direct, face-to-face interactions between people, causing college students to alienate themselves from real-world interpersonal interactions to a certain extent, and affecting the improvement of interpersonal communication capacity [62].

### 4.3. Communication Capacity Was Negatively Correlated with Social Anxiety

According to previous studies, maladaptive behavior and irrational cognitive perceptions are two important causes of social anxiety among college students [20]. Behaviorism suggests that, as an emotional response, social anxiety stems from conditioned effects and explains the formation of social anxiety through the principle of conditioned effects and social learning theory [63]. That is, social anxiety can arise from a lack of social skills. The stronger the individual’s communication capacity, the lower the level of social anxiety. A possible explanation for this is that the greater one’s communication capacity is, the more clearly one can communicate one’s wishes and ideas to others, and the more acutely one can detect the subtle emotional feelings of others through their body language. Hence, one tends to build up a good level of confidence when interacting socially with others, which, as a good emotional experience, helps to reduce the occurrence of social anxiety [64]. On the other hand, people with lower communication capacity may have a poorer sense of communication experience in everyday interpersonal interactions due to their lack of essential communication skills, and are therefore more reluctant to engage in frequent interactions with people. The less they communicate with people, the more they fear communicating with people, thus increasing their level of social anxiety [65].

### 4.4. The Mediating Role of Communication Capacity

The other results of this study corroborate the theoretical validity of the mediation model; communication capacity partially mediates the relationship between social media use and social anxiety, which means that communication capacity represents a potential underlying mechanism that could partially explain how social media use is linked with social anxiety. That is, promoting positive social media use and decreasing passive social media use as ways to build up communication capacity might help to relieve social anxiety among college students. Specifically, active use of social media strengthens relational connections between individuals and provides a supportive environment for improving communication capacity [66], thus helping to reduce social anxiety. On the other hand, passive social media use significantly increases the risk of developing social anxiety, which can be buffered by enhancing communication capacity. Behaviorism also suggests that social anxiety can be generated by a lack of social skills [63]. Therefore, the development of interventions oriented towards enhancing the communication capacity of college students is crucial today, when social networks are prevalent. This may help these individuals to expand their social communication resources and strengthen their interpersonal support, thereby reducing social anxiety.

This study has a few limitations. First, while this study was limited to public colleges, there were some differences in students’ use of social media across different types of schools. To improve the generalizability of the results, future studies could replicate this study in other educational institutions (e.g., private or international schools). Second, our mediation model is based on a priori, derived from previous studies. However, it is only one of several reasonable and possible models examining how different variables are related. Future research needs to consider the mediating role of other variables not studied in this research, and verify whether the outcomes are replicated at other educational levels. Finally, while the findings of this study support the hypothesized relationships described in the existing literature, additional prospective studies are required to confirm the results.

## 5. Conclusions

Our research extends the previous results, showing that the relationship between social media use and social anxiety can be explained when incorporating communication capacity as a mediator. Active social media use was significantly and negatively related to social anxiety, whereas passive social networking site use was significantly and positively related to social anxiety. Reducing the use of passive social media among college students and adopting communication capacity-oriented interventions may yield benefits for improving students’ psychological wellbeing; educators should pay sufficient attention to them.

## Figures and Tables

**Table 1:** Participants’ sociodemographic characteristics (N = 1740).

Characteristics	N (%)	Social Anxiety		
		M ± SD	t/F	*p*
Age (year)		19.43 ± 1.85		
Gender			−0.359	*p* < 0.001
Male	679 (39.0%)	42.63 ± 8.64		
Female	1061 (61.0%)	44.00 ± 8.09		
Only child in family		43.10 ± 8.48	−1.583	0.114
Yes	749 (43.0%)	43.74 ± 8.22		
No	991 (57.0%)			
Grade		43.65 ± 8.04	0.186	0.906
Freshman	565 (32.5%)	43.41 ± 8.53		
Sophomore	600 (34.5%)	43.20 ± 9.43		
Junior	239 (13.7%)	43.19 ± 7.58		
Senior or above	317 (19.3%)	43.43 ± 7.65		
Place of origin				
City area	863 (49.6%)	43.04 ± 8.42	−2.127	0.054
Rural area	877 (50.4%)	43.88 ± 8.24		
Monthly per capita household income				
<3000 yuan	256 (14.7%)	44.56 ± 7.54	5.595	*p* < 0.001
3000–5000 yuan	644 (37.0%)	44.35 ± 8.35		
5000–7000 yuan	516 (29.7%)	43.11 ± 7.92		
>7000 yuan	324 (18.6%)	42.35 ± 8.43		
Teaching assistants experience			−0.762	0.446
Yes	1035 (59.48%)	43.34 ± 8.53		
No	705 (40.52%)	43.65 ± 8.04		
Ethnic minorities				
Yes	38 (2.2%)	42.54 ± 6.96	0.545	0.586
No	1702 (97.8%)	43.30 ± 8.10		
Childhood left-behind experience			2.462	0.014
Yes	55 (3.2%)	46.18 ± 8.62		
No	1685 (96.8%)	43.38 ± 8.31		
Single parent families				
Yes	114 (6.6%)	44.48 ± 8.98	1.350	0.117
No	1626 (93.4%)	43.39 ± 8.28		
Frustration experience in social interactions			65.589	*p* < 0.001
Very often	186 (10.7%)	49.02 ± 9.06		
General	719 (41.3%)	44.14 ± 6.94		
Occasionally	609 (35.0%)	42.88 ± 7.94		
None	226 (13.0%)	38.30 ± 9.54		
Number of friends on SNSs			1.297	0.269
<100	223 (12.8%)	42.55 ± 8.93		
100–300	616 (35.4%)	43.86 ± 8.02		
301–500	739 (42.5%)	43.51 ± 8.24		
501–700	76 (4.4%)	42.46 ± 8.59		
>700	86 (4.9%)	43.47 ± 9.44		
EPQ-Personality traits			59.527	*p* < 0.001
Introverted	889 (51.1%)	45.18 ± 7.60		
Intermediate	729 (41.9%)	41.97 ± 7.81		
Extroverted	122 (7.0%)	37.64 ± 7.51		

Notes: SNSs: Social networking sites; EPQ: Eysenck Personality Questionnaire.

**Table 2:** Correlation analysis of social media use, communication capacity, and social anxiety (r).

Item	1	2	3	4	5	6	7	8	9	10	11	12
Passive social media use	1											
Active social media use	0.019	1										
Communication capacity	−0.253 **	0.514 **	1									
Verbal expression	−0.229 **	0.452 **	0.648 **	1								
Enthusiasm	−0.213 **	0.505 **	0.599 **	0.534 **	1							
Emotional sensitivity	−0.192 **	0.251 **	0.710 **	0.533 **	0.515 **	1						
Comforting others	−0.137 **	0.488 **	0.669 **	0.551 **	0.559 **	0.587 **	1					
Respect	−0.170 *	0.350 **	0.689 **	0.440 **	0.494 **	0.714 **	0.536 **	1				
Empathy	0.093 **	0.517 **	0.709 **	0.525 **	0.496 **	0.680 **	0.604 **	0.766 **	1			
Listening	−0.165	0.390 **	0.613 **	0.441 **	0.542 **	0.521 **	0.548 **	0.633 **	0.641 **	1		
Emotional control	−0.379 **	0.572 **	0.703 **	0.303 **	0.234 **	0.269 **	0.262 **	0.223 **	0.247 **	0.284 **	1	
Social anxiety	0.525 **	−0.342 **	−0.371 **	−0.330 **	0.237	−0.342	−0.386 **	0.212	−0.230	0.353 *	−0.479 **	1

Note: * *p* < 0.05, ** *p* < 0.01.

**Table 3:** Structural equation model fit index.

Fit Index	CMIN/*df*	CFI	GFI	AGFI	RMSEA
Test result	2.121	0.975	0.975	0.998	0.089
Fit standard	1 < *χ*^2^/*df* < 3	>0.90	>0.90	>0.90	<0.08

Note: CMIN/DF, Chi-square minimum degrees of freedom; CFI, comparative fit index; GFI, goodness of fit index; AGFI, adjusted goodness of fit index; RMSEA, root mean square error of approximation.

**Table 4:** Bootstrap analysis of the mediating effect of communication ability between social media use and social anxiety.

Path	Effect	Normalized Path Coefficient (β)	Standard Error (S.E.)	95% CI	*p*
1. Passive social media use → Communication capacity → Social anxiety	Direct effect (c’)	0.646	0.059	0.577~0.715	<0.001
	Indirect effect (ab)	0.050	0.013	0.007~0.059	0.003
	Total effect (c)	0.696	0.031	0.636~0.757	<0.001
2. Active social media use → Communication capacity → Social anxiety	Direct effect (c’)	−0.477	0.039	−0.552~−0.401	<0.001
	Indirect effect (ab)	−0.100	0.014	−0.086~−0.030	<0.001
	Total effect (c)	−0.576	0.033	−0.641~−0.512	<0.001

## Data Availability

The data presented in this study are available on reasonable request from the corresponding author. The data are not publicly available due to ethical requirements.

## Appendix A. Items of Communication Capacity Scale

Respect	I can respect others in terms of manners.
	I can accommodate other people’s perspectives.
	I speak politely.
Listening	I’m a good listener.
	I listen to others carefully when I talk to them.
	I can’t concentrate on listening to others.
Empathy	I can accurately understand the thoughts of others, whether they are elders or peers.
	When I disagree with my family, I will think from a different perspective and work together to solve the problem.
	I can put myself in others’ shoes.
Emotional sensitivity	I can easily perceive the emotional feelings of others.
	I can perceive social situations well and pay attention to what others say and do.
	I can interpret other people’s attitudes and expressions based on their gestures, expressions, or eyes.
Comforting others	I like to comfort others.
	I think it is useless to comfort others when they are in trouble.
	I am good at comforting others when they encounter misfortune or difficulties.
	When friends feel upset or angry, they are willing to talk to me.
Emotional control	I can find many reasonable ways to deal with my negative emotions without causing harm to myself or others.
	It’s very difficult for me to control my emotions.
	When someone misunderstands me, I can explain to him/her calmly
Enthusiasm	I appear to be cold.
	I will take the initiative to say hello when I meet people I know.
	I don’t initiate communication with new acquaintances.
	I am an enthusiastic person.
	I always smile with people.
Verbal expression	I can express my thoughts clearly.
	I can describe the boring things vividly.
	To make my speech more compelling, I incorporate gestures and facial expressions.
	People always comprehend what I’m saying easily.
	I know how to change the subject and understand the main points of the conversation.
	I can control my nerves in front of strangers and converse happily with them.
	I don’t speak fluently.
Note: Only the items for the eight dimensions are shown here, and the scale’s 7 polygraph questions have been omitted.	

## References

[1] Scott G.G., Boyle E.A., Czerniawska K., Courtney A. (2018). Posting Photos on Facebook: The Impact of Narcissism, Social Anxiety, Loneliness, and Shyness. Personal. Individ. Differ..

[2] Goel A., Jaiswal A., Manchanda S., Gautam V., Aneja J., Raghav P. (2020). Burden of Internet Addiction, Social Anxiety and Social Phobia among University Students, India. J. Fam. Med. Prim Care.

[3] Honnekeri B.S., Goel A., Umate M., Shah N., De Sousa A. (2017). Social Anxiety and Internet Socialization in Indian Undergraduate Students: An Exploratory Study. Asian J. Psychiatry.

[4] Russell G., Shaw S. (2009). A Study to Investigate the Prevalence of Social Anxiety in a Sample of Higher Education Students in the United Kingdom. J. Ment. Health.

[5] Feng F., Wang C., Wang Y., Hu S., Shi H. (2018). An investigation study on anxiety and depression among college students in medical schools and analysis of its causes. J. Hebei Med. Univ..

[6] Fink M., Akimova E., Spindelegger C., Hahn A., Lanzenberger R., Kasper S. (2009). Social Anxiety Disorder: Epidemiology, Biology and Treatment. Psychiatr. Danub..

[7] Çimke S., Cerit E. (2021). Social Media Addiction, Cyberbullying and Cyber Victimization of University Students. Arch. Psychiatr. Nurs..

[8] Vannucci A., Flannery K.M., Ohannessian C.M. (2017). Social Media Use and Anxiety in Emerging Adults. J. Affect. Disord..

[9] Schlenker B.R., Leary M.R. (1982). Social Anxiety and Self-Presentation: A Conceptualization Model. Psychol. Bull..

[10] Pettigrew T.F., Suls J.M., Miller R.L. (1979). Social Comparison Processes: Theoretical and Empirical Perspectives. Contemp. Sociol..

[11] de Vries D.A., Peter J., de Graaf H., Nikken P. (2015). Adolescents’ Social Network Site Use, Peer Appearance-Related Feedback, and Body Dissatisfaction: Testing a Mediation Model. J. Youth Adolesc..

[12] Soohinda G., Ojha K., Sampath H., Dutta S. (2021). Social Networking Sites and Its Relation to Social Comparison and Psychological Well-Being among Medical University Students. Indian J. Psychiatry.

[13] Mukhopadhyay D., Barman L., Bandyopadhyay G. (2018). Use of Social Networking Site and Mental Disorders among Medical Students in Kolkata, West Bengal. Indian J. Psychiatry.

[14] Yu L., Du M. (2022). Social Networking Use, Mental Health, and Quality of Life of Hong Kong Adolescents during the COVID-19 Pandemic. Front. Public Health.

[15] Mauri M., Cipresso P., Balgera A., Villamira M., Riva G. (2011). Why Is Facebook So Successful? Psychophysiological Measures Describe a Core Flow State While Using Facebook. Cyberpsychol. Behav. Soc. Netw..

[16] Rose C.A., Tynes B.M. (2015). Longitudinal Associations Between Cybervictimization and Mental Health Among U.S. Adolescents. J. Adolesc. Health.

[17] Chen W., Lee K.-H. (2013). Sharing, Liking, Commenting, and Distressed? The Pathway Between Facebook Interaction and Psychological Distress. Cyberpsychol. Behav. Soc. Netw..

[18] Deters F.G., Mehl M.R. (2012). Does Posting Facebook Status Updates Increase or Decrease Loneliness? An Online Social Networking Experiment. Soc. Psychol. Personal. Sci..

[19] Verduyn P., Lee D.S., Park J., Shablack H., Orvell A., Bayer J., Ybarra O., Jonides J., Kross E. (2015). Passive Facebook Usage Undermines Affective Well-Being: Experimental and Longitudinal Evidence. J. Exp. Psychol. Gen..

[20] Schønning V., Hjetland G.J., Aarø L.E., Skogen J.C. (2020). Social Media Use and Mental Health and Well-Being Among Adolescents—A Scoping Review. Front. Psychol..

[21] Guetterman T.C., Sakakibara R., Baireddy S., Kron F.W., Scerbo M.W., Cleary J.F., Fetters M.D. (2019). Medical Students’ Experiences and Outcomes Using a Virtual Human Simulation to Improve Communication Skills: Mixed Methods Study. J. Med. Internet Res..

[22] Wong H.Y., Mo H.Y., Potenza M.N., Chan M.N.M., Lau W.M., Chui T.K., Pakpour A.H., Lin C.-Y. (2020). Relationships between Severity of Internet Gaming Disorder, Severity of Problematic Social Media Use, Sleep Quality and Psychological Distress. Int. J. Environ. Res. Public Health.

[23] Gong R., Zhang Y., Long R., Zhu R., Li S., Liu X., Wang S., Cai Y. (2021). The Impact of Social Network Site Addiction on Depression in Chinese Medical Students: A Serial Multiple Mediator Model Involving Loneliness and Unmet Interpersonal Needs. Int. J. Environ. Res. Public Health.

[24] Li J.-B., Feng L.-F., Wu A.M.S., Mai J.-C., Chen Y.-X., Mo P.K.H., Lau J.T.F. (2021). Roles of Psychosocial Factors on the Association Between Online Social Networking Use Intensity and Depressive Symptoms Among Adolescents: Prospective Cohort Study. J. Med. Internet Res..

[25] Halls G., Cooper P.J., Creswell C. (2015). Social Communication Deficits: Specific Associations with Social Anxiety Disorder. J. Affect. Disord..

[26] Verduyn P., Ybarra O., Résibois M., Jonides J., Kross E. (2017). Do Social Network Sites Enhance or Undermine Subjective Well-Being? A Critical Review. Soc. Issues Policy Rev..

[27] Solmi F., Bentivegna F., Bould H., Mandy W., Kothari R., Rai D., Skuse D., Lewis G. (2020). Trajectories of Autistic Social Traits in Childhood and Adolescence and Disordered Eating Behaviours at Age 14 Years: A UK General Population Cohort Study. J. Child Psychol. Psychiatr..

[28] Stednitz J.N., Epkins C.C. (2006). Girls’ and Mothers’ Social Anxiety, Social Skills, and Loneliness: Associations After Accounting for Depressive Symptoms. J. Clin. Child Adolesc. Psychol..

[29] Çunkuş N., Yiğitoğlu G.T., Solak S. (2021). The Relationship between Worry and Comfort Levels and Communication Skills of Nursing Students during Pediatric Clinic Applications: A Descriptive Study. Nurse Educ. Today.

[30] Oh M., Laugeson E., Kim J.-H., Lee K., Kim J., Lee S., Lim B., Cha S., Bong G., Yoon N.-H. (2021). A Randomized Controlled Trial of the Korean Version of the Program for the Education and Enrichment of Relational Skills for Young Adults (PEERS®-YA-K) With Autism Spectrum Disorder: A Pilot Study. Front. Psychiatry.

[31] Frison E., Eggermont S. (2015). Exploring the Relationships Between Different Types of Facebook Use, Perceived Online Soci-al Support, and Adolescents’ Depressed Mood. Soc. Sci. Comput. Rev..

[32] Tandoc E.C., Ferrucci P., Duffy M. (2015). Facebook Use, Envy, and Depression among College Students: Is Facebooking Depressing?. Comput. Hum. Behav..

[33] LIU Q., NIU G., Fan C., ZHOU Z. (2017). Passive Use of Social Network Site and Its Relationships with Self-Esteem and Self-Concept Clarity: A Moderated Mediation Analysis. Acta Psychol. Sin..

[34] Jin L., Jian C. (2012). A study on the relationship between interpersonal efficacy and communication skills of college students. J. Neijiang Norm. Coll..

[35] Leary M.R. (1983). Social Anxiousness: The Construct and Its Measurement. J. Personal. Assess..

[36] Min Q., Guo W., Rong Z., Zi Z. (2000). Revision of the Chinese version of the Eysenck Personality Questionnaire Short Form Scale (EPQ-RSC). J. Psychol..

[37] Eysenck S.B.G., Eysenck H.J., Barrett P. (1985). A Revised Version of the Psychoticism Scale. Personal. Individ. Differ..

[38] Costache M.E., Frick A., Månsson K., Engman J., Faria V., Hjorth O., Hoppe J.M., Gingnell M., Frans Ö., Björkstrand J. (2020). Higher- and Lower-Order Personality Traits and Cluster Subtypes in Social Anxiety Disorder. PLoS ONE.

[39] Fang K., Friedlander M., Pieterse A.L. (2016). Contributions of Acculturation, Enculturation, Discrimination, and Personality Traits to Social Anxiety among Chinese Immigrants: A Context-Specific Assessment. Cult. Divers. Ethn. Minor. Psychol..

[40] Podsakoff P.M., MacKenzie S.B., Podsakoff N.P. (2012). Sources of Method Bias in Social Science Research and Recommendations on How to Control It. Annu. Rev. Psychol..

[41] Hayes A. (2013). Introduction to Mediation, Moderation, and Conditional Process Analysis: A Regression-Based Approach. J. Educ. Meas..

[42] Hu L., Bentler P.M. (1999). Cutoff Criteria for Fit Indexes in Covariance Structure Analysis: Conventional Criteria versus New Alternatives. Struct. Equ. Model. A Multidiscip. J..

[43] Henricks L.A., Pouwels J.L., Lansu T.A.M., Lange W., Becker E.S., Klein A.M. (2021). Prospective Associations between Social Status and Social Anxiety in Early Adolescence. Br. J. Dev. Psychol..

[44] Tang X., Liu Q., Cai F., Tian H., Shi X., Tang S. (2022). Prevalence of Social Anxiety Disorder and Symptoms among Chi-nese Children, Adolescents and Young Adults: A Systematic Review and Meta-Analysis. Front. Psychol..

[45] Cross S.E., Madson L. (1997). Models of the Self: Self-Construals and Gender. Psychol. Bull..

[46] Markus H.R., Kitayama S. (1991). Culture and the Self: Implications for Cognition, Emotion, and Motivation. Psychol. Rev..

[47] Bai C., Chen X., Liu H., Yu K. (2022). Parental Expectation and Mobile Phone Addiction in Adolescents from Chinese Low-Income Families: The Mediating Effects of Self-Esteem and Social Anxiety. Cyberpsychol. Behav. Soc. Netw..

[48] Jefferies P., Ungar M. (2020). Social Anxiety in Young People: A Prevalence Study in Seven Countries. PLoS ONE.

[49] Norton A.R., Abbott M.J. (2017). The Role of Environmental Factors in the Aetiology of Social Anxiety Disorder: A Review of the Theoretical and Empirical Literature. Behav. Chang..

[50] Abdollahi A., Ahmed A.A.A., Suksatan W., Kumar T., Majeed M.S., Zainal A.G., Dokoushkani F., Allen K.A. (2022). Courage: A Potential Mediator of the Relationship Between Personality and Social Anxiety. Psychol. Stud..

[51] Acarturk C., Cuijpers P., van Straten A., de Graaf R. (2008). Psychological Treatment of Social Anxiety Disorder: A Meta-Analysis. Psychol. Med..

[52] Kaplan S.C., Levinson C.A., Rodebaugh T.L., Menatti A., Weeks J.W. (2015). Social Anxiety and the Big Five Personality Traits: The Interactive Relationship of Trust and Openness. Cogn. Behav. Ther..

[53] Costa P.T., McCrae R.R. (1992). Normal Personality Assessment in Clinical Practice: The NEO Personality Inventory. Psychol. Assess..

[54] Bienvenu O.J., Hettema J.M., Neale M.C., Prescott C.A., Kendler K.S. (2007). Low Extraversion and High Neuroticism as Indices of Genetic and Environmental Risk for Social Phobia, Agoraphobia, and Animal Phobia. Am. J. Psychiatry.

[55] Cohen J. (2022). Statistical Power Analysis for the Behavioral Sciences. The SAGE Encyclopedia of Research Design.

[56] Dienlin T., Johannes N. (2020). The Impact of Digital Technology Use on Adolescent Well-Being. Dialogues Clin. Neurosci..

[57] Ng Y.-L. (2019). Active and Passive Facebook Use and Associated Costly Off-Line Helping Behavior. Psychol. Rep..

[58] Steinfield C., Ellison N.B., Lampe C. (2008). Social Capital, Self-Esteem, and Use of Online Social Network Sites: A Longitudinal Analysis. J. Appl. Dev. Psychol..

[59] Burke M., Marlow C., Lento T. (2010). Social Network Activity and Social Well-Being. Proceedings of the 28th International Conference-E on Human Factors in Computing Systems (CHI’10).

[60] Ding Q., Zhang Y.-X., Wei H., Huang F., Zhou Z.-K. (2017). Passive Social Network Site Use and Subjective Well-Being among Chinese University Students: A Moderated Mediation Model of Envy and Gender. Personal. Individ. Differ..

[61] Xiao Y., Jian C., Ci L. (2021). The relationship between stress perception and loneliness among senior college students: A cross-lagged analysis. Neurol. Disord. Ment. Health.

[62] Shuai L., Yuan T., Xiao S., Chen Z. (2017). The relationship between proactive social networking site use and adolescent friendship quality: The mediating role of positive feedback and interpersonal uncertainty. Psychol. Behav. Res..

[63] Jones F.N., Skinner B.F. (1939). The Behavior of Organisms: An Experimental Analysis. Am. J. Psychol..

[64] Pickard H., Rijsdijk F., Happé F., Mandy W. (2017). Are Social and Communication Difficulties a Risk Factor for the Devel-opment of Social Anxiety?. J. Am. Acad. Child Adolesc. Psychiatry.

[65] Poole K.L., Van Lieshout R.J., Schmidt L.A. (2017). Exploring Relations between Shyness and Social Anxiety Disorder: The Role of Sociability. Personal. Individ. Differ..

[66] Liu D., Brown B.B. (2014). Self-Disclosure on Social Networking Sites, Positive Feedback, and Social Capital among Chinese College Students. Comput. Hum. Behav..

